# Which aspects of continuity of care in primary care are associated with better self-rated health? A cross-sectional study among older adults in Berlin

**DOI:** 10.1186/s12875-026-03566-1

**Published:** 2026-09-24

**Authors:** Yana Zhou, Marie Bolster, Denis Gerstorf, Raphael Kohl, Dagmar Haase, Manuel Wolff, Angelique Chan, Paul Gellert, Wolfram J. Herrmann

**Affiliations:** 1 Institute of General Practice and Family Medicine, Charité - Universitätsmedizin Berlin, corporate member of Freie Universität Berlin and Humboldt Universität zu Berlin, Charitéplatz 1, Berlin, 10117 Germany; 2 Department of Psychology, Humboldt-Universität zu Berlin, Unter den Linden 6, Berlin, 10099 Germany; 3 Deutsches Institut für Wirtschaftsforschung (DIW), Socio-Economic Panel, Berlin, 10117 Germany; 4 Institute of Medical Sociology and Rehabilitation Science, Charité - Universitätsmedizin Berlin, corporate member of Freie Universität Berlin and Humboldt-Universität zu Berlin, Charitéplatz 1, Berlin, 10117 Germany; 5 Geography Department, Humboldt-Universität zu Berlin, Unter den Linden 6, Berlin, 10099 Germany; 6 Department of Computational Landscape Ecology, Helmholtz Centre for Environmental Research - UFZ, Permoserstraße 15, Leipzig, 04318 Germany; 7 Centre for Ageing Research and Education, Duke-NUS Medical School, Singapore, Singapore; 8 Einstein Center Population Diversity (ECPD), Berlin, Germany

**Keywords:** Continuity of care, Self-rated health, Primary care, Older adults, Urban health, Germany

## Abstract

**Background:**

Global population ageing and rapid urbanization are increasing the prevalence of multimorbidity and complex health needs among older adults, emphasizing the importance of accessible and high-quality primary care. Continuity of care (CoC) is a core feature of person-centered primary care and has been linked to improved health outcomes, reduced mortality, and lower health care utilization. While general use of primary care has been associated with better self-rated health, evidence on the specific role of continuity of primary care remains limited. This study examined the association between CoC in primary care and self-rated health among adults aged 65 years and older living in three neighborhoods in Berlin, Germany.

**Methods:**

Cross-sectional data were drawn from the baseline survey of the *Ageing Well* study (December 2023 – June 2024, December 2024 – June 2025) in Berlin. 837 participants completed standardized face-to-face interviews. CoC was assessed by duration of care at a general practitioner’s practice and duration of care with the same general practitioner. Self-rated health was measured using a single-item five-point scale. Linear regression models were fitted, adjusting for age, gender, subjective social status, lifetime morbidity, symptoms of depression, and anxiety. Missing data were addressed using listwise deletion.

**Results:**

Most participants reported a general practitioner as their usual care site (84.7%), and self-rated health was most frequently rated as good (41.6%). Continuity with a general practitioner was positively associated with self-rated health, although the magnitude of the association was small (b = 0.008, 95% CI: 0.002–0.015). Lifetime morbidity explained the largest proportion of unique variance in self-rated health. Depressive symptoms were associated with poorer self-rated health, while higher subjective social status was associated with better self-rated health.

**Conclusions:**

The association between CoC and self-rated health was small. These findings suggest that commonly used patient-reported outcomes may not adequately capture the effects of CoC in older adults. Future research should therefore consider more sensitive and process-oriented outcome measures to better capture the impact of CoC.

**Clinical trial number:**

German Register for Clinical Studies (DRKS) (DRKS00033043, registered 16 November 2023); part of the WHO International Clinical Trials Registry Platform (WHO ICTRP), https://www.drks.de.

**Supplementary Information:**

The online version contains supplementary material available at https://doi.org/10.1186/s12875-026-03566-1.

## Background

Global trends show that the population aged over 65 is growing faster than any other age group [1] with an estimated number over 1 billion, and more than half of the global population expected to reside in urban areas within the next twenty years [2]. Increasing longevity is often associated with increases in multiple chronic conditions and complex health needs, highlighting the importance of accessible, high-quality primary care [3]. A well-established primary care system is fundamental to improving overall health characterized by fewer chronic illnesses and increased life expectancy [4].

A fundamental feature of primary care, continuity of care (CoC) is emphasized by the World Health Organization as central to integrated, people-centered health services [5–7]. Three types of CoC can be differentiated: informational, management, and relational [8]. This study focuses on relational continuity, which refers to the sustained relationship between a patient and a healthcare provider, thereby promoting mutual understanding and alignment of care with patients’ preferences and priorities [9–11].

High levels of CoC have been associated with lower healthcare expenditures and improved system efficiency [12], as well as enhanced care quality [13], greater patient satisfaction [14], and more effective prioritization of health needs [15]. Moreover, CoC has been linked to reduced mortality in North America [9, 16] and Europe [11, 17]. Older adults with higher CoC also tend to have fewer emergency department visits [3, 18, 19] and hospitalizations [18–22]. Research in Taiwan further shows that CoC can improve medication adherence and prevent duplicate prescriptions [23, 24], and has been associated with a reduced risk of suicide [25]. Continuity is generally stronger in practices led by independent physicians compared to resident clinics [26].

As CoC has been shown to have several positive effects on healthcare outcomes and tends to increase with age [27], it is important to identify health outcomes that are particularly relevant for healthy ageing [28], especially from the patient´s perspective. Patient-reported outcome measures (PROMs) are important to address individual health care needs appropriately, facilitating, for example, shared decision making between patients and clinicians [29]. Self-rated health, a widely recognized PROM of overall health [30], is an essential component of healthy ageing assessment [31]. Poor self-rated health is linked to chronic conditions [32], unmet health needs [33], physical inactivity [34], low educational attainment [35] and mental health issues [36, 37]. Self-rated health also predicts mortality, morbidity [38] and the onset of conditions such as stroke [39], with factors such as age [40], education [41], income [36], subjective social status [42] and gender [40, 43] influencing these outcomes.

Although prior research indicates that general use of primary care may improve self-rated health [44], the specific contribution of continual use of primary care remains contradictory and underexplored. While evidence from Sub-Saharan Africa indicates that higher relational continuity is associated with better self-rated health [43], other studies found no association between CoC and self-rated health [45, 46].

This study therefore aims to address this gap of knowledge in the German healthcare context by investigating the relationship between continuity of primary care and self-rated health among older adults in Berlin. We hypothesize that if there is an association between continuity of care and self-rated health, it would be reflected in having a regular general practitioner (GP) and in a longer duration of care with their provider.

## Method

### Study design and sample

The study employed a cross-sectional design. Data were derived from the baseline survey of the study *“Ageing Well in the Urban Environment: Meeting the Health and Social Needs of Older Adults”* at the Berlin study site, for which a detailed study description has been published previously [47]. The English version of the questionnaire is provided in Additional file 1.

The sample comprised 837 randomly selected older adults from three neighborhoods (spanning seven so-called life-space oriented areas) in Berlin, which were Marzahn-Hellersdorf, Spandau and Reinickendorf. Potential participants were randomly selected from the registry offices. Eligibility criteria included being 65 years or older, being registered as a resident in one of the three neighborhoods and not having a legal guardian. After providing written consent, standardized computer-assisted personal interviews (CAPIs) were conducted with participants via home visit by trained interviewers. Functional measures (cognitive test, grip strength, and walking speed) were assessed as part of the interview. Data collection of the baseline survey took place between December 2023 and June 2024 and between December 2024 and June 2025.

### Measures

#### Independent variable

Continuity of primary care was assessed based on the continuity of care model [48], which has been applied in the National Health and Health Services Use Questionnaire (NHHSUQ). Continuity of care was initially assessed across different usual care providers, including general practitioners, other outpatient care, emergency departments and nonspecific care places, as in the model of Bentler et al. [48]. However, the distribution of responses across these categories was highly uneven, with the majority of participants reporting a usual general practitioner, while only a small number reported continuity with another usual care provider. Due to the limited number of observations in these categories, including them in regression analyses would have resulted in unstable estimates. Therefore, the present analysis focuses on continuity with general practitioners.

The assessment in the present study focuses on longitudinal continuity and comprises two key dimensions: general practitioner site continuity and general practitioner doctor continuity (Table 1).

**Table 1:** Operationalization of continuity of care measures

Dimension	Question	Response options
GP site continuity	“Approximately how long have you been receiving care at this place?”	0 = other care site/no usual care site 0.25 = < 6 months 0.75 = 6 months to < 1 year 1.5 = 1 to < 2 years 2.5 = 2 to < 5 years 5 = ≥ 5 years
GP doctor continuity	“How long have you visited this care provider?”	continuous (years) 0 = other care site/no usual care provider

*GP* General practitioner

The dimension of GP site continuity was measured using predefined categories reflecting increasing lengths of engagement with the same GP practice. Participants with other or no usual care site were coded as zero years of GP site continuity to allow their inclusion in the analysis. This approach was chosen because the number of participants in the non-GP categories was too small to permit meaningful separate analyses.

The GP doctor continuity dimension was assessed by asking participants about the length of their relationship with their general practitioner, if they had indicated that they typically consult a general practitioner. Participants who reported another usual care provider or no usual care provider were coded as having zero years of GP doctor continuity. The same coding approach was applied to GP doctor continuity because the number of participants in the non-GP categories was too small to permit meaningful separate analyses.

Variance Inflation Factor (VIF) was used to test for multicollinearity of the CoC variables, which was < 1.5.

#### Dependent variable

Subjective health status was assessed using a single-item self-rating question: “In general, would you describe your state of health as excellent, very good, good, moderate, or poor?” (1 = poor, 2 = moderate, 3 = good, 4 = very good, 5 = excellent).

### Covariates

To account for potential confounding, several covariates were included. Sociodemographic variables included *age* (calculated from registry-based date of birth) and *self-reported gender*. We measured *subjective social status* (SSS) with the German Version of the MacArthur Scale (ranging from 1 = lowest to 10 = highest) [49], which shows stable reliability and construct validity [50, 51]. *Lifetime morbidity* was measured by asking for lifetime diagnoses by a medical professional using a list of 26 chronic conditions based on the Simple Segmentation Tool [52]. Participants were asked if they had ever been diagnosed with one of 26 chronic conditions. A prorated lifetime morbidity score was calculated for participants with missing values on individual morbidity items, provided that at least 75% of the items were completed. For eligible participants, the number of endorsed items was divided by the number of completed items and subsequently multiplied by the total number of items, thereby scaling the observed sum score to the full item set. The resulting score could theoretically range from 0 to 26, reflecting the estimated number of lifetime diagnoses across the assessed conditions. Participants with more than 25% missing values on the morbidity items were excluded from analyses involving this variable. To assess the robustness of our findings, we conducted a sensitivity analysis using an alternative operationalization of morbidity. In addition to the prorated morbidity score, we calculated a morbidity ratio by dividing the number of reported diagnoses by the number of items answered. *Mental health* was assessed using the Patient Health Questionnaire-4 (PHQ-4). A cut-off score of ≥ 3 on the respective subscales was used to indicate current symptoms of depression or anxiety [53].

### Statistical analysis

To investigate the association between continuity of care and self-rated health, we conducted linear regression analyses. Self-rated health was treated as a continuous outcome variable. Although self-rated health is measured on an ordinal five-point scale, previous studies have suggested that treating ordered response categories as an approximately continuous measure can preserve information from the original categories and facilitate statistical analyses and interpretation of differences in self-rated health across groups [54]. Three regression models were specified with stepwise adjustments. Akaike information criterion (AIC) and adjusted R² were used to identify the best fit. Model 1 included GP site continuity and GP doctor continuity. Model 2 additionally adjusted for sociodemographic covariates including age, gender, and subjective social status. Model 3 further included clinical covariates (lifetime morbidity, symptoms of depression, and symptoms of anxiety). To evaluate the robustness of the findings, we repeated the fully adjusted regression model after excluding participants with GP doctor continuity values exceeding 40 years (*n* = 9). This analysis examined whether the observed association between GP doctor continuity and self-rated health was driven by a small number of very high continuity values. In additional exploratory analyses, the three neighborhoods were included as effect-coded predictors to examine potential neighborhood differences and interactions with continuity of care in relation to self-rated health. Missing data were handled using listwise deletion, as the proportion of missing values across all model variables was below 5%. To assess the potential for selection bias, participants included in the analyses were compared with those excluded because of missing data. Wilcoxon rank-sum tests were used for continuous and ordinal variables, and Fisher’s exact tests were used for categorical variables. Regression coefficients are reported as unstandardized estimates (b) with 95% confidence intervals to facilitate interpretation in the original units of self-rated health. Effect sizes were quantified using partial R² to assess the proportion of variance uniquely explained by each predictor. Partial R² values were derived from the corresponding t statistics. All statistical analyses were conducted with R version 4.4.1 [55]. Statistical significance was defined at *p* <.05.

## Results

### Sample characteristics

The total sample included 837 participants. One participant had missing data on self-rated health, therefore, analyses stratified by self-rated health were based on 836 participants. Among these participants, age ranged from 65 to 100 years (M = 75.74, SD = 6.90), and 60.8% were women. Lifetime morbidity ranged from 0 to 16.25 chronic conditions (M = 4.78, SD = 2.81). Most participants reported a general practitioner as their usual care site (84.6%), and 66.42% had attended their general practitioners practice for five years or more. The mean duration of the relationship with the same general practitioner was 7.41 years (SD = 10.05), ranging from 0 to 55 years. Self-rated health was most frequently reported as “good” (41.6%), with a mean score of 2.65 (SD = 0.92). Sample characteristics stratified by self-rated health are presented in Table 2.

**Table 2:** Sample characteristics by self-rated health

Characteristic	Self-rated health					
	Excellent	Very good	Good	Moderate	Poor	Total
Self-rated health (*n* = 836)	25 (2.99)	103 (12.32)	348 (41.63)	278 (33.25)	82 (9.81)	836 (100)
Age (*n* = 836)	71.68 (5.53)	73.74 (6.42)	75.53 (6.86)	76.99 (6.93)	76.15 (6.95)	75.74 (6.90)
Age group (*n* = 836)						
65–69 years	10 (40.00)	32 (31.07)	76 (21.84)	39 (14.03)	16 (19.51)	173 (20.69)
70–74 years	8 (32.00)	30 (29.13)	107 (30.75)	69 (24.82)	23 (28.05)	237 (28.35)
75–79 years	5 (20.00)	19 (18.45)	60 (17.24)	66 (23.74)	15 (18.29)	165 (19.74)
80–84 years	1 (4.00)	16 (15.53)	66 (18.97)	65 (23.38)	13 (15.85)	161 (19.26)
85 + years	1 (4.00)	6 (5.83)	39 (11.21)	39 (14.03)	15 (18.29)	100 (11.96)
Gender (*n* = 832)						
Female	12 (48.00)	65 (63.11)	218 (62.82)	167 (60.51)	44 (54.32)	506 (60.82)
Male	13 (52.00)	38 (36.89)	129 (37.18)	109 (39.49)	37 (45.68)	326 (39.18)
Neighborhood (*n* = 836)						
Marzahn-Hellersdorf	8 (32.00)	38 (36.89)	142 (40.80)	96 (34.53)	25 (30.49)	309 (36.96)
Reinickendorf	9 (36.00)	35 (33.98)	100 (28.74)	108 (38.85)	27 (32.93)	279 (33.37)
Spandau	8 (32.00)	30 (29.13)	106 (30.46)	74 (26.62)	30 (36.59)	248 (29.67)
SSS (*n* = 830)	7.56 (1.64)	7.14 (1.62)	6.55 (1.45)	6.33 (1.67)	5.84 (1.83)	6.51 (1.63)
Lifetime morbidity (*n* = 833)	2.52 (2.22)	2.81 (1.81)	4.12 (2.41)	5.86 (2.63)	7.12 (3.04)	4.78 (2.81)
Symptoms of depression (*n* = 821)						
No	25 (100.00)	101 (98.06)	332 (97.08)	246 (90.44)	60 (75.95)	764 (93.06)
Yes	0 (0.00)	2 (1.94)	10 (2.92)	26 (9.56)	19 (24.05)	57 (6.94)
Symptoms of anxiety (*n* = 822)						
No	25 (100.00)	102 (99.03)	329 (96.20)	254 (93.04)	68 (86.08)	778 (94.65)
Yes	0 (0.00)	1 (0.97)	13 (3.80)	19 (6.96)	11 (13.92)	44 (5.35)
Usual care site (*n* = 833)						
General practitioner	23 (92.00)	90 (87.38)	296 (85.30)	233 (84.42)	63 (76.83)	705 (84.63)
Other outpatient care	1 (4.00)	5 (4.85)	22 (6.34)	26 (9.42)	7 (8.54)	61 (7.32)
Emergency department	0 (0.00)	0 (0.00)	2 (0.58)	3 (1.09)	2 (2.44)	7 (0.84)
Other, not specified	0 (0.00)	1 (0.97)	9 (2.59)	6 (2.17)	3 (3.66)	19 (2.28)
No usual care site	1 (4.00)	7 (6.80)	18 (5.19)	8 (2.90)	7 (8.54)	41 (4.92)
GP site continuity (*n* = 810)						
Other care place	2 (8.00)	12 (11.88)	43 (12.84)	35 (13.11)	17 (21.52)	109 (13.51)
Less than 6 months	0 (0.00)	1 (0.99)	3 (0.90)	1 (0.37)	1 (1.27)	6 (0.74)
6 months till 1 year	1 (4.00)	4 (3.96)	12 (3.58)	6 (2.25)	3 (3.80)	26 (3.22)
1 year to 2 years	2 (8.00)	6 (5.94)	16 (4.78)	9 (3.37)	1 (1.27)	34 (4.21)
2 years to 5 years	4 (16.00)	12 (11.88)	46 (13.73)	25 (9.36)	9 (11.39)	96 (11.90)
5 years or more	16 (64.00)	66 (65.35)	215 (64.18)	191 (71.54)	48 (60.76)	536 (66.42)
GP doctor continuity (*n* = 800)	8.33 (9.45)	8.88 (12.59)	7.28 (9.89)	7.15 (9.83)	6.76 (7.84)	7.41 (10.05)

Values are presented as mean (SD) for continuous variables and n (%) for categorical variables. Table values are based on participants with available self-rated health data (*n* = 836). Variable-specific sample sizes are indicated after the variable name. Percentages are calculated based on available data

*M* Mean, *SD* Standard deviation, *SSS* Subjective social status, *Lifetime morbidity* Mean of chronic conditions with SD, *GP* General practitioner

### Linear regression

Regression results are presented in Table 3.

**Table 3:** Linear regression of self-rated health

Predictor		Model 1		Model 2		Model 3	
	b	95% CI	b	95% CI	b	95% CI	Partial *R*²
GP site continuity	−0.006	−0.043, 0.03	−0.016	−0.052, 0.02	−0.021	−0.054, 0.012	0.002
GP doctor continuity	0.005	−0.002, 0.012	0.006	0, 0.013	0.008**	0.002, 0.015	0.009
Age			−0.018***	−0.028, −0.009	−0.01*	−0.019, −0.002	0.008
Gender (male)			−0.013	−0.143, 0.117	−0.13*	−0.25, −0.009	0.006
SSS			0.127***	0.088, 0.166	0.054**	0.017, 0.092	0.011
Lifetime morbidity					−0.132***	−0.154, −0.11	0.157
Symptoms of depression					−0.396**	−0.651, −0.142	0.013
Symptoms of anxiety					−0.134	−0.416, 0.149	0.001
*n*	775		769		748		
R²	0.002		0.071		0.244		
Adjusted R²	0		0.065		0.236		
AIC	2082.01		2013.8		1807.97		

Partial *R²* values are reported as measures of effect size. Significance levels correspond to the t-test of the regression coefficients.

*b* Unstandardized regression coefficient, 95% *CI* 95% confidence interval, *GP* General practitioner, *SSS* Subjective social status, *AIC* Akaike information criterion

*p <.05, **p <.01, *** *p* <.001

Model 3 demonstrated the lowest Akaike Information Criterion (AIC) and the highest adjusted R² and was therefore selected as the final model. Among the continuity of care measures, GP doctor continuity was associated with higher self-rated health (b = 0.008), whereas GP site continuity was not significantly associated with self-rated health. Regarding covariates, older age (b = − 0.01) and male gender (b = − 0.13) were both associated with lower self-rated health. Subjective social status showed a positive association (b = 0.054), while lifetime morbidity (b = − 0.132) and symptoms of depression (b = − 0.396) were negatively associated with self-rated health. Symptoms of anxiety were not significantly associated with self-rated health. The final model explained 23.6% of the variance in self-rated health. Among the significant predictors, lifetime morbidity explained the largest proportion of unique variance in self-rated health (partial R² = 0.157). Results of the sensitivity analysis using an alternative operationalization of morbidity were consistent across both measures, with effect estimates in the same direction and statistically significant (Table 4, Additional file 2). The sensitivity analysis excluding participants with GP doctor continuity values exceeding 40 years yielded comparable results to the main analysis, with the association between GP doctor continuity and self-rated health remaining statistically significant (b = 0.01, 95% CI: 0.003–0.017, Table 5, Additional file 3). In the exploratory analysis, no significant interaction effects were observed between continuity of care measures and neighborhood (Table 6, Additional file 4). Finally, participants included in the regression analyses were compared with those excluded due to missing data. A significant difference was found only for GP doctor continuity (*p* <.001, Table 7, Additional file 5).

## Discussion

### Summary

This cross-sectional study examined the relationship between continuity of care and self-rated health among adults aged 65 years and older in Berlin. Most participants reported good self-rated health and identified a general practitioner as their usual care site. Regression analyses distinguished between two dimensions of continuity. Duration of care at the GP practice level was not significantly associated with self-rated health, whereas longer continuity with the same individual GP showed a statistically significant association. However, the effect size was small and of questionable clinical relevance. Among all covariates, lifetime morbidity explained the largest proportion of unique variance in self-rated health.

The prevalence of good self-rated health in this sample was broadly consistent with international evidence from comparable age groups [56]. However, a healthy participant effect is likely to have introduced some upward bias in self-rated health, as individuals with severe illness may have been unable or unwilling to participate. This suggests that the true burden of poor self-rated health in this age group may be underestimated in the present study, and findings should be interpreted with this limitation in mind.

Overall, the findings suggest that the association between continuity of care and self-rated health was small. While this contrasts with Kasenda et al. [43], who reported positive associations between relational continuity and self-rated health, it is consistent with other investigations that found no significant relationship [45, 46]. Previous research in older adults with multiple chronic conditions reported no association between CoC and self-rated health over time [45]. One explanation for these findings is that self-rated health, although a well-established and validated patient-reported outcome, may not be sufficiently sensitive to capture the effects of CoC in older populations. Continuity represents only one component of a complex and multifactorial care process, whereas self-rated health reflects a broad range of biological, psychological, and social influences. As such, potential benefits of continuity may not translate into measurable differences in global health assessments. This interpretation is supported by a systematic review by Worrall and Knight [46], showing that improvements in care delivery may enhance coordination, trust, and patient experience, without necessarily affecting overall health ratings. Therefore, the absence of an observable association should not be interpreted as evidence that continuity of care is unimportant, but rather that its effects may not be adequately captured by the outcome measures used in this study.

In addition, opposing mechanisms may have attenuated observable associations. Individuals with poorer health may remain longer within the same care setting due to ongoing treatment needs and more frequent consultations [57]. In contrast, individuals with better health may require fewer contacts with health care providers and may therefore be less likely to maintain long-term relationships with a specific practice. These countervailing processes may have attenuated any observable association between duration of care at the GP practice level and self-rated health.

Lifetime morbidity accounted for the largest proportion of unique variance in self-rated health, consistent with previous research linking multimorbidity to poorer self-rated health [32, 37]. It should be noted that lifetime morbidity does not distinguish between conditions that are currently active and those that have resolved. However, the strong negative association with self-rated health suggests that a substantial proportion of the reported conditions are likely chronic in nature and therefore still clinically relevant at the time of assessment. This is consistent with the understanding that many common morbidities, such as cardiovascular disease, diabetes, or musculoskeletal disorders, tend to persist over time and continue to influence individuals’ perception of their health, which may also underpin the assumption for individuals with poorer health to remain longer within the same care setting.

Male gender was associated with poorer self-rated health. This finding contrasts with previous studies that reported poorer self-rated health among women [43]. One possible explanation for this unexpected finding may be differences in utilization of general practitioner services. However, this hypothesis was not assessed in the present study and therefore remains speculative.

Higher subjective social status was associated with better self-rated health, consistent with existing literature [42]. Since both are self-reported measures, shared method variance cannot be ruled out as a contributing factor. However, the association between SES and self-rated health is well-established across studies using objective SES indicators as well [41], suggesting that the present finding reflects a genuine relationship. Previous research further suggests that this association may be mediated by social trust, whereby higher SSS is linked to greater social trust, which in turn is associated with better self-rated health [56, 58, 59].

Depressive symptoms were significantly associated with poorer self-rated health, whereas anxiety did not show an independent association. This is consistent with studies demonstrating strong links between depression and self-rated health [36, 37, 40, 41]. Depression may be more strongly related to self-rated health because its symptoms directly affect health perception, and overall well-being. As the PHQ-4 subscales for depression and anxiety show substantial intercorrelation [60], the independent association of anxiety may have been attenuated in the multivariable model due to shared variance with depression. Whether anxiety has a distinct effect on self-rated health beyond its overlap with depression remains an open question for future research.

Age was associated with self-rated health, supporting prior studies suggesting a decline in self-rated health with increasing age due to the cumulative burden of morbidity [40].

Additional exploratory analyses did not provide evidence that the association between continuity of care and self-rated health differed across residential contexts. This suggests that the observed relationship, particularly for GP doctor continuity, may be relatively robust across different neighborhood settings.

### Strength and limitations

This study is among the first to investigate the relationship between continuity of primary care and self-rated health in Germany, providing novel insights into this underexplored context. It included adults aged 65 and above from three socially and demographically diverse districts in Berlin, enhancing sample heterogeneity within an urban setting. Data collection through home visits enabled the inclusion of individuals with limited mobility and reduced exclusion due to accessibility barriers. The use of structured, interviewer-administered questionnaires supported data quality and completeness, and random selection of participants helped to reduce selection bias. A key strength of this study is the use of validated instruments. Continuity of care was measured using an established tool [48] facilitating comparability with previous and future studies. Self-rated health, a widely used and validated indicator of perceived health [38, 61], provided a reliable outcome measure. Ethical standards and data protection requirements were strictly adhered to throughout the study.

In closing, we note several limitations of our study. The cross-sectional design precludes causal inference, limiting interpretation to associations. Data were self-reported, introducing potential reporting and recall bias. Simultaneous interviewing of spouses or partners may have influenced responses. Selection bias may have occurred, as older, sicker, or lower-SSS individuals, as well as non-German speakers, were likely underrepresented, potentially excluding particularly vulnerable migrant populations. In addition, missing data were handled using listwise deletion. Although participants excluded because of missing data differed significantly from those included with regard to GP doctor continuity, no significant differences were observed for the remaining study variables. Therefore, some selection bias due to missing data cannot be ruled out. Residual confounding cannot be ruled out despite adjustment for key variables, including age, gender, subjective social status, lifetime morbidity, and symptoms of depression and anxiety. Although self-rated health is a widely used and validated global measure of perceived health [38, 61], its assessment using a single-item measure may not capture all dimensions contributing to perceived health status, which could have influenced the observed associations. Continuity of care was analyzed only for general practitioners. Participants who reported another usual care provider or no usual care provider were coded as having zero years of GP continuity because the number of respondents in these categories was too small for meaningful separate analyses. Consequently, the operationalization of GP continuity may not distinguish between the absence of GP continuity and continuity of care provided by other healthcare professionals, such as specialists. Focusing on general practitioners reflects the predominant source of care but limits the assessment of broader continuity of care. Therefore, the findings should be interpreted as reflecting continuity with a general practitioner rather than continuity of care more broadly. Furthermore, given the study focus on relational continuity of care, continuity of care was operationalized using the duration of the relationship with a general practitioner. Although duration captures an important aspect of relational continuity, this measure may not fully reflect the complexity of the construct and may therefore have attenuated the observed associations. In addition, participants may have had difficulty distinguishing between continuity with an individual GP and continuity with a practice, particularly within the German primary care system, where care is often shared among multiple physicians and patients are free to change providers. This is supported by reported durations of GP continuity of up to 55 years, suggesting that some participants may have referred to the practice rather than an individual physician. As these values were not isolated outliers and could not be definitively classified as erroneous, they were retained in the analysis. Future research should use longitudinal designs, provide multilingual materials, and integrate administrative data to better capture different dimensions of continuity and their association with patient-related health outcomes.

## Conclusions

In conclusion, this study found only a small association between continuity with an individual GP and self-rated health among older adults in Berlin, while no association was observed for continuity at the GP practice level. While our findings may imply that there are further interacting factors that explain the relation between healthcare and self-rated health, this absence of clear findings should not be taken to imply that continuity of care lacks relevance, but rather that its effects may lie in more specific outcomes that are not captured by broad measures such as self-rated health. Instead, continuity of care may primarily influence process-oriented outcomes, including patient experience and trust, which have been highlighted in previous qualitative research [62, 63] but remain insufficiently captured in quantitative analyses. Future research in the German healthcare context should therefore focus on these outcomes and apply longitudinal or administrative data to better capture the impact of continuity over time. Strengthening the conceptual and methodological alignment between continuity measures and outcome indicators will be essential for advancing evidence in this field.

## Supplementary Information

Additional file 1: English version of the questionnaire. File format: .docx. Description: This file contains the English version of the questionnaire “Ageing Well in the Urban Environment: Meeting the Health and Social Needs of Older Adults”.

Additional file 2: Table 4. Linear regression of self-rated health with alternative morbidity score. File format: .docx. Description: This file contains the table with results of the linear regression of model 3 of self-rated health with alternative morbidity score.

Additional file 3: Table 5. Linear regression of self-rated health excluding participants with GP doctor continuity > 40 years. File format: .docx. Description: This file contains the table with the results of the linear regression of self-rated health excluding participants with GP doctor continuity >40 years.

Additional file 4: Table 6. Linear regression of self-rated health with effect-coded predictor neighborhood. File format: .docx. Description: This file contains the table with the results of the linear regression of model 3 of self-rated health with effect-coded predictor neighborhood.

Additional file 5: Table 7. Comparison of participants included and excluded in the final linear regression model due to missing data. File format: .docx. Description: This file contains the table with the results of the comparison of participants included and excluded in the final linear regression model due to missing data.

## Data Availability

The data underlying this article will be shared on reasonable request to the corresponding author. However, it will only be shared in part, as the written consent included an opt-in/opt-out option and some participants chose not to have their data included in the open data set.

## Abbreviations

CoC: Continuity of care
95% CI: 95% confidence interval
SRH: Self-rated health
GP: General practitioner
DRKS: German Register for Clinical Studies
PROM: Patient related outcome measure
CAPI: Computer-assisted personal interviews
NHHSUQ: National Health and Health Services Use Questionnaire
VIF: Variance inflation factor
SSS: Subjective social status
SES: Socioeconomic status
PHQ-4: Patient Health Questionnaire-4
AIC: Akaike information criterion
