# Supplementary material for: Which aspects of continuity of care in primary care are associated with better self-rated health? A cross-sectional study among older adults in Berlin
Source: BMC Prim Care. 2026 Sep 24;27:339. doi: 10.1186/s12875-026-03566-1 (PMC13602587; doi:10.1186/s12875-026-03566-1)
Supplement: Supplementary file 1 [file 12875_2026_3566_MOESM1_ESM.docx]

**Ageing Well in the Urban Environment**:

**Meeting the Health and Social Needs of Older Adults**

**BASELINE MAIN QUESTIONNAIRE**

| **SECTION A: HOUSEHOLD AND FAMILY COMPOSITION** | | | | | | | | | |
| --- | --- | --- | --- | --- | --- | --- | --- | --- | --- |
| **To begin with, we would like to ask you a few questions about your household.** | | | | | | | | | |
| **A1** | **What type of accommodation do you live in?** | | | | | | | | |
|  | 1 | Apartment (rented) | | | | | | | |
|  |  | Which floor is the apartment on? _________ | | | | | | | |
|  | 2 | Apartment (owned) | | | | | | | |
|  |  | Which floor is the apartment on? _________ | | | | | | | |
|  | 3 | Detached house/semi-detached house (rented) | | | | | | | |
|  |  | Is there an elevator? | 1 | Yes | | 2 | No | 777 | Refused |
|  | 4 | Detached house/semi-detached house (owned) | | | | | | | |
|  |  | Is there an elevator? | 1 | Yes | | 2 | No | 777 | Refused |
|  | 5 | Other, please specify: _________ | | | | | | | |
|  | 999 | Don’t know | 777 | | Refused | | | | |
| **A2** | **About how long have you lived in this current house?** | | | | | | | | |
|  | _________ years _________ months | | | | | | | | |
|  | 999 | Don’t know | 777 | | Refused | | | | |
| **A3** | **How many persons live in your household?**  **Please include yourself, domestic worker (maid), those who live here but are temporarily hospitalised, children, and household members who are currently in National Service / Reservist training. Please exclude any tenants who live in your household.** | | | | | | | | |
|  | Number of persons in household including subject: _________ | | | | | | | | |
|  | 999 | Don’t know | 777 | | Refused | | | | |
| **The next questions are about your family.** | | | | | | | | | |
| **A4** | **What is your current marital status?** | | | | | | | | |
|  | 1 | Married (includes being separated from the spouse due to spouse being hospitalised, living in an institution, or living in another area for business reasons or to take care of others) | | | | | | | |
|  | 2 | In a partnership | | | | | | | |
|  | 3 | Widowed | | | | | | | |
|  | 4 | Separated from spouse | | | | | | | |
|  | 5 | Divorced | | | | | | | |
|  | 6 | Single | | | | | | | |
|  | 999 | Don’t know | 777 | | Refused | | | | |
| **A5** | **How many surviving children do you have? Please include biological/natural and adopted/stepchildren?** | | | | | | | | |
|  | Number of children: _____________ | | | | | | | | |
|  | 999 | Don’t know | 777 | | Refused | | | | |

| **SECTION B: LONELINESS** | | | | | | | | |
| --- | --- | --- | --- | --- | --- | --- | --- | --- |
| **Below, you will find a series of statements about social relationships. Please indicate the extent to which you agree or disagree with each statement, using the following scale: “strongly disagree” to “strongly agree.” You may use the options in between to reflect varying degrees of agreement.** | | | | | | | | |
|  | | **Strongly Disagree** | **Disagree** | **Neither Agree nor Disagree** | **Agree** | **Strongly Agree** | **Don’t Know** | **Refused** |
| **B1** | I lack companionship. | 1 | 2 | 3 | 4 | 5 | 999 | 777 |
| **B2** | There are people I feel close to. | 1 | 2 | 3 | 4 | 5 | 999 | 777 |
| **B2** | There are people I can turn to. | 1 | 2 | 3 | 4 | 5 | 999 | 777 |
| **B4** | I feel isolated. | 1 | 2 | 3 | 4 | 5 | 999 | 777 |
| **B5** | I feel part of a group of friends/acquaintances. | 1 | 2 | 3 | 4 | 5 | 999 | 777 |
| **B6** | There are people I can talk to openly. | 1 | 2 | 3 | 4 | 5 | 999 | 777 |
| **B7** | I feel left out. | 1 | 2 | 3 | 4 | 5 | 999 | 777 |
| **B8** | I feel lonely. | 1 | 2 | 3 | 4 | 5 | 999 | 777 |

| **SECTION C: RISK OF SOCIAL ISOLATION (Lubben Social Network Short Scale – 6)** | | | | | | | | | |
| --- | --- | --- | --- | --- | --- | --- | --- | --- | --- |
| **Considering the people to whom you are related by birth, marriage, adoption, etc…** | | | | | | | | | |
|  | | **Number of relatives** | | | | | | **Don’t know** | **Refused** |
| **C1** | How many relatives do you see or hear from at least once a month? | 0 | 1 | 2 | 3-4 | 5-8 | ≥9 | 999 | 777 |
| **C2** | How many relatives do you feel at ease with that you can talk about private matters? | 0 | 1 | 2 | 3-4 | 5-8 | ≥9 | 999 | 777 |
| **C3** | How many relatives do you feel close to such that you could call on them for help? | 0 | 1 | 2 | 3-4 | 5-8 | ≥9 | 999 | 777 |
| **Considering all of your friends including those who live in your neighbourhood** | | | | | | | | | |
|  | | **Number of friends** | | | | | | **Don’t know** | **Refused** |
| **C4** | How many of your friends do you see or hear from at least once a month? | 0 | 1 | 2 | 3-4 | 5-8 | ≥9 | 999 | 777 |
| **C5** | How many friends do you feel at ease with that you can talk about private matters? | 0 | 1 | 2 | 3-4 | 5-8 | ≥9 | 999 | 777 |
| **C6** | How many friends do you feel close to such that you could call on them for help? | 0 | 1 | 2 | 3-4 | 5-8 | ≥9 | 999 | 777 |

| **SECTION D: NEIGHBOURHOOD SOCIAL COHESION** | | | | | | | | |
| --- | --- | --- | --- | --- | --- | --- | --- | --- |
| **I will now ask you about the context of your local area that is everywhere within a 20-minute walk or about a kilometer of your home. To what extent do you agree or disagree with the following statements?** | | | | | | | | |
|  | | **Strongly Disagree** | **Disagree** | **Neither Agree nor Disagree** | **Agree** | **Strongly Agree** | **Don’t Know** | **Refused** |
| **D1** | I really feel part of  this area | 1 | 2 | 3 | 4 | 5 | 999 | 777 |
| **D2** | Most people in this area can be trusted | 1 | 2 | 3 | 4 | 5 | 999 | 777 |
| **D3** | People feel safe walking alone in this area after dark | 1 | 2 | 3 | 4 | 5 | 999 | 777 |
| **D4** | Most people in this area are friendly | 1 | 2 | 3 | 4 | 5 | 999 | 777 |
| **D5** | This area is kept very clean | 1 | 2 | 3 | 4 | 5 | 999 | 777 |
| **D6** | If you were in trouble, there are lots of people in this area who would help you | 1 | 2 | 3 | 4 | 5 | 999 | 777 |
| **D7** | There is no problem with vandalism and graffiti in this area | 1 | 2 | 3 | 4 | 5 | 999 | 777 |
| **D8** | There are no vacant houses or storefronts in this area | 1 | 2 | 3 | 4 | 5 | 999 | 777 |
| **SECTION E: THE AGE-FRIENDLY ENVIRONMENTAL ASSESSMENT TOOL (AFEAT)** | | | | | | | | |
| **When answering each statement consider how easy/difficult it is to access each resource based on current physical and cognitive capabilities. To what extent do you agree or disagree with the following statements?** | | | | | | | | |
|  | | **Strongly Disagree** | **Disagree** | **Neither Agree nor Disagree** | **Agree** | **Strongly Agree** | **Don’t Know** | **Refused** |
| **E1** | I am able to ac­cess coffeshops, hawker centres or restaurants without any issue | 1 | 2 | 3 | 4 | 5 | 999 | 777 |
| **E2** | I am able to access shops, supermarkets or wetmarkets without any issue | 1 | 2 | 3 | 4 | 5 | 999 | 777 |
| **E3** | I am able to access local services such as shops, restaurants, maintenance services, or GP clinics without any issue. | 1 | 2 | 3 | 4 | 5 | 999 | 777 |
| **E4** | I am able to access General practitioner (GP) clinics (i.e., private doctor clinics in the neighbourhood) or polyclinics without any issue | 1 | 2 | 3 | 4 | 5 | 999 | 777 |
| **E5** | I am able to walk around the local area/community without problems. | 1 | 2 | 3 | 4 | 5 | 999 | 777 |
| **E6** | I am able to take public transport such as buses or MRT or LRT, without problems | 1 | 2 | 3 | 4 | 5 | 999 | 777 |
| **E7** | I live in close proximity to local services such as shops, restaurants, maintenance services, and GP clinics. | 1 | 2 | 3 | 4 | 5 | 999 | 777 |
| **E8** | I live in close proximity to public green spaces, such as parks, gardens, woodlands. | 1 | 2 | 3 | 4 | 5 | 999 | 777 |
| **E9** | My housing is safe, clean and well-maintained. | 1 | 2 | 3 | 4 | 5 | 999 | 777 |
| **E10** | There are plenty of places to meet up with friends and family, as well as engage in community activities. | 1 | 2 | 3 | 4 | 5 | 999 | 777 |
| **E11** | I am able to access Active Ageing Centres or Senior Care Centres without any issue | 1 | 2 | 3 | 4 | 5 | 999 | 777 |
| **E12** | There is consistent outreach to include people at risk of social isolation. | 1 | 2 | 3 | 4 | 5 | 999 | 777 |
| **E13** | I feel I am a valuable part of my local community. | 1 | 2 | 3 | 4 | 5 | 999 | 777 |
| **E14** | I can engage in volunteer or paid work without worrying about any special requirements I may have. | 1 | 2 | 3 | 4 | 5 | 999 | 777 |
| **E15** | I have easy access to information regarding the local community and I am able to give my opinion on community-based decision making. | 1 | 2 | 3 | 4 | 5 | 999 | 777 |
| **E16** | I have easy access to information and services regarding my health. | 1 | 2 | 3 | 4 | 5 | 999 | 777 |

| **SECTION F: LIFE SPACE ASSESSMENT** | | | | | | | | | |
| --- | --- | --- | --- | --- | --- | --- | --- | --- | --- |
| **Following questions refer to your activities over the past 4 weeks:** | | | |  | | | | **How did you get there?** | |
| **During the past 4 weeks, have you been to (F1-F5)?** | | | | **How often have you been there?** | | | | **Did you use aids or equipment?** | **Did you need help from another person?** |
| **F1** | **Other rooms of your home besides the room where you sleep? *This includes areas within your home such as other rooms, kitchen, living room, toilet.*** | Yes | No | Less than 1 time/ week | 1-3 times/week | 4-6 times/week | Daily | ☐Yes  ☐No  ☐Don’t know/refused | ☐Yes  ☐No  ☐ Don’t know/refused |
|  |  | ☐[1] | ☐[0] | ☐[1] | ☐[2] | ☐[3] | ☐[4] |  |  |
|  | *Score* | ________ x | | ___________ x | | | | _____ = _____ | |
| **F2** | **An area outside your home that is still adjacent to your home? *This includes places immediately outside your home such as rubbish chute, lift, stairway, or corridor (e.g., to water plants).*** | Yes | No | Less than 1 time/ week | 1-3 times/week | 4-6 times/week | Daily | ☐Yes  ☐No  ☐Don’t know/refused | ☐Yes  ☐No  ☐Don’t know/refused |
|  |  | ☐[1] | ☐[0] | ☐[1] | ☐[2] | ☐[3] | ☐[4] |  |  |
|  | *Score* | ________ x | | ___________ x | | | | _____ = _____ | |
| **F3** | **Places in your immediate neighbourhood? *This includes areas outside your home, surrounding your home, such as void deck, carpark, playground, exercise corner, or coffeeshop.*** | Yes | No | Less than 1 time/ week | 1-3 times/week | 4-6 times/week | Daily | ☐Yes  ☐No  ☐Don’t know/refused | ☐Yes  ☐No  ☐Don’t know/refused |
|  |  | ☐[1] | ☐[0] | ☐[1] | ☐[2] | ☐[3] | ☐[4] |  |  |
|  | *Score* | ________ x | | ___________ x | | | | _____ = _____ | |
| **F4** | **Places outside your immediate neighbourhood, but within your town?** | Yes | No | Less than 1 time/ week | 1-3 times/week | 4-6 times/week | Daily | ☐Yes  ☐No  ☐Don’t know/refused | ☐Yes  ☐No  ☐Don’t know/refused |
|  |  | ☐[1] | ☐[0] | ☐[1] | ☐[2] | ☐[3] | ☐[4] |  |  |
|  | *Score* | ________ x | | ___________ x | | | | _____ = _____ | |
| **F5** | **Places outside your town?** | Yes | No | Less than 1 time/ week | 1-3 times/week | 4-6 times/week | Daily | ☐Yes  ☐No  ☐Don’t know/refused | ☐Yes  ☐No  ☐Don’t know/refused |
|  |  | ☐[1] | ☐[0] | ☐[1] | ☐[2] | ☐[3] | ☐[4] |  |  |
|  | *Score* | ________ x | | ___________ x | | | | _____ = _____ | |

**SECTION G: DIGIT SUBSTITUTION TEST**

Note for interviewers: The DSST is administered on paper. Please follow the instructions on the DSST handout.

| **SECTION H: HEALTHCARE** | | | | |
| --- | --- | --- | --- | --- |
| **Next, we would like to ask you a few questions about healthcare.** | | | | |
| **H1** | **What type of health insurance or healthcare coverage do you have?** | | | |
|  | 1 | Statutory health insurance (“Gesetzliche Krankenversicherung”) | | |
|  | 2 | Private health insurance (including civil service aid scheme / “Beihilfe”).  This does not refer to supplementary private insurance in addition to statutory health insurance | | |
|  | 3 | Other entitlement to healthcare (e.g. special public healthcare coverage such as for police or military personnel, or foreign health insurance) | | |
|  | 4 | No health insurance (self-paying) | | |
|  | 999 | Don’t know | 777 | Refused |
| **The following questions relate to consultations with your general practitioner (GP) or family doctor.**  **Please include visits to the doctor’s office, home visits, and telephone consultations.** | | | | |
| **H2** | **How often have you consulted a general practitioner (GP) or family doctor for yourself in the past 12 months?** | | | |
|  | _________ times (0 if none) | | | |
|  | 999 | Don’t know | 777 | Refused |
| **The following questions relate to consultations with medical or surgical specialists. Please include only outpatient or emergency consultations, but not contacts with doctors during an inpatient or day-patient hospital stay.** | | | | |
| **H3** | **How often have you consulted a medical or surgical specialist for yourself in the past 12 months?** | | | |
|  | _________ times (0 if none) | | | |
|  | 999 | Don’t know | 777 | Refused |
| **H4** | **Have you visited an emergency department for yourself in the past 6 months?** | | | |
|  | 1 | Yes | 2 | No |
|  | 999 | Don’t know | 777 | Refused |
| **H5** | **Have you been hospitalized in the past 6 months (i.e., stayed at least one night in a hospital)?** | | | |
|  | 1 | Yes | 2 | No |
|  | 999 | Don’t know | 777 | Refused |
| **13 Item Continuity of Care model as used in the NHHSUQ (National health and health services use questionnaire)** | | | | |
| **Longitudinal continuity: care site** | | | | |
| **H6** | **Of the places you go for medical care, where do you go most often for care if you are sick or need advice about your health?** | | | |
|  | 4 | Primary physician/GP | | |
|  | 3 | Other outpatient care | | |
|  | 2 | Emergency room or urgent care | | |
|  | 1 | Other, nonspecific | | |
|  | 0 | No specific place | | |
|  | 999 | Don’t know | 777 | Refused |
| **H7** | **How long have you been receiving your care at this place?** | | | |
|  | 0,25 | Less than 6 months | | |
|  | 0,75 | 6 months to 1 year | | |
|  | 1,5 | 1 year to 2 years | | |
|  | 2,5 | 2 years to 5 years | | |
|  | 5 | 5 years or more | | |
|  | 0 | No specific care place | | |
|  | 999 | Don’t know | 777 | Refused |
| **Longitudinal continuity: provider duration** | | | | |
| **H8** | **Is there a specific doctor you usually visit as part of your regular medical care?** | | | |
|  | 1 | Yes | 2 | No |
|  | 999 | Don’t know | 777 | Refused |
| **H9** | **How long have you been a patient of this doctor?** | | | |
|  | _________ years | | | |
|  | 999 | Don’t know | 777 | Refused |
| **H10** | **Have you changed this doctor within the past 12 months?** | | | |
|  | 1 | Yes | 2 | No |
|  | 999 | Don’t know | 777 | Refused |

| **SECTION I: HEALTH STATUS** | | | | |
| --- | --- | --- | --- | --- |
| **The next set of questions is about your health.** | | | | |
| **I1** | **In general, would you describe your state of health as excellent, very good, good, fair or poor?** | | | |
|  | 1 | Excellent | | |
|  | 2 | Very Good | | |
|  | 3 | Good | | |
|  | 4 | Fair | | |
|  | 5 | Poor | | |
|  | 999 | Don’t Know | 777 | Refused |
| **I2** | **The next question is related to your vision. With your glasses or contact lenses if you wear them, is your eyesight excellent, very good, good, fair, poor, or do you have loss of vision in both eyes?** | | | |
|  | 1 | Excellent | | |
|  | 2 | Very good | | |
|  | 3 | Good | | |
|  | 4 | Fair | | |
|  | 5 | Poor | | |
|  | 6 | Loss of vision (no vision) in both eyes | | |
|  | 999 | Don’t Know | 777 | Refused |
| **I3** | **The next question is related to your hearing ability. If you use hearing aids, please respond to this question based on your hearing ability when you wear them. Is your hearing excellent, very good, good, fair, poor or are you not able to hear in both ears?** | | | |
|  | 1 | Excellent | | |
|  | 2 | Very good | | |
|  | 3 | Good | | |
|  | 4 | Fair | | |
|  | 5 | Poor | | |
|  | 6 | Not able to hear in both ears | | |
|  | 999 | Don’t Know | 777 | Refused |

| **SECTION K: SIMPLE SEGMENTATION TOOL – COMMUNITY VERSION** | | | | | | | | | | | |
| --- | --- | --- | --- | --- | --- | --- | --- | --- | --- | --- | --- |
| **The following questions relate to your medical history.** | | | | | | | | | | | |
|  | **Have you ever been diagnosed by a medical professional with…** | | | | | | **Yes** | **No** | **Don’t know** | | **Refused** |
| **K1** | Coronary artery disease (such as angina or heart attack) | | | | | | 1 | 2 | 999 | | 777 |
| **K2** | Heart failure | | | | | | 1 | 2 | 999 | | 777 |
| **K3** | Other forms of heart diseases | | | | | | 1 | 2 | 999 | | 777 |
| **K4** | Cancer | | | | | | 1 | 2 | 999 | | 777 |
| **K5** | Cerebrovascular disease (such as stroke) | | | | | | 1 | 2 | 999 | | 777 |
| **K6** | High blood pressure or Hypertension | | | | | | 1 | 2 | 999 | | 777 |
| **K7** | High blood sugar or Diabetes | | | | | | 1 | 2 | 999 | | 777 |
| **K8** | High blood cholesterol or lipids | | | | | | 1 | 2 | 999 | | 777 |
| **K9** | Chronic respiratory illness (e.g., asthma or COPD [chronic obstructive pulmonary disease]) | | | | | | 1 | 2 | 999 | | 777 |
| **K10** | Chronic digestive illness (stomach or intestinal) (e.g., Gastro-esophageal reflux disease [GERD], inflammatory bowel disease [IBD]-Crohn's disease, irritable bowel syndrome [IBS]) | | | | | | 1 | 2 | 999 | | 777 |
| **K11** | Chronic renal / kidney or urinary tract ailments (e.g., chronic kidney disease [CKD], renal/ kidney failure) | | | | | | 1 | 2 | 999 | | 777 |
| **K12** | Chronic ailments of the liver or gall bladder (e.g., hepatitis B, liver cirrhosis) | | | | | | 1 | 2 | 999 | | 777 |
| **K13** | Joint pain, arthritis, rheumatism or nerve pain | | | | | | 1 | 2 | 999 | | 777 |
| **K14** | Chronic back pain | | | | | | 1 | 2 | 999 | | 777 |
| **K15** | Osteoporosis | | | | | | 1 | 2 | 999 | | 777 |
| **K16** | Fractures of the hip, thigh or pelvis | | | | | | 1 | 2 | 999 | | 777 |
| **K17** | Glaucoma | | | | | | 1 | 2 | 999 | | 777 |
| **K18** | Epilepsy | | | | | | 1 | 2 | 999 | | 777 |
| **K19** | Age-related macular degeneration | | | | | | 1 | 2 | 999 | | 777 |
| **K20** | Autoimmune disorder | | | | | | 1 | 2 | 999 | | 777 |
| **K21** | Chronic skin condition (e.g., eczema, psoriasis) | | | | | | 1 | 2 | 999 | | 777 |
| **K22** | Thyroid disorder | | | | | | 1 | 2 | 999 | | 777 |
| **K23** | Migraine | | | | | | 1 | 2 | 999 | | 777 |
| **K24** | Parkinson’s Disease | | | | | | 1 | 2 | 999 | | 777 |
| **K25** | Depression | | | | | | 1 | 2 | 999 | | 777 |
| **K26** | Anxiety disorder | | | | | | 1 | 2 | 999 | | 777 |
| **The next few questions are about your healthcare utilization for these health conditions that you have mentioned.** | | | | | | | | | | | |
| **K27** | **For each condition, how many times did you go to the emergency department (A&E) in the past six months?**  *[Instruction for Interviewer:* Ask about each condition individually that was reported in response to the question above*.]* | | | | | | | | | | |
|  |  | | | *____ times* | 999 | Don’t know | | | 777 | Refused | |
|  |  | | | *____ times* | 999 | Don’t know | | | 777 | Refused | |
|  |  | | | *____ times* | 999 | Don’t know | | | 777 | Refused | |
|  |  | | | *____ times* | 999 | Don’t know | | | 777 | Refused | |
|  |  | | | *____ times* | 999 | Don’t know | | | 777 | Refused | |
|  | *[If necessary, additional “ID” rows can be added in Qualtrics.]* | | | | | | | | | | |
| **K28** | **How many hospital stays have you had in connection with each condition (i.e. where you stayed at least one night in hospital)?**  **Please include hospital admissions where you were admitted directly to hospital, as well as those where you first attended a hospital emergency department and were then admitted to hospital from there.** | | | | | | | | | | |
|  |  | | | *____ times* | 999 | Don’t know | | | 777 | Refused | |
|  |  | | | *____ times* | 999 | Don’t know | | | 777 | Refused | |
|  |  | | | *____ times* | 999 | Don’t know | | | 777 | Refused | |
|  |  | | | *____ times* | 999 | Don’t know | | | 777 | Refused | |
|  |  | | | *____ times* | 999 | Don’t know | | | 777 | Refused | |
|  | *[If necessary, additional “ID” rows can be added in Qualtrics.]* | | | | | | | | | | |
| **K29** | **Were you admitted to a hospital for any other reasons in the past 6 months? If so, how many times?** | | | | | | | | | | |
|  | 1 | Yes, *____ times* | | | | | | | | | |
|  | 2 | No | | | | | | | | | |
|  | 999 | Don’t know | 777 | Refused | | | | | | | |
| **K30** | **During the past 6 months, which of the following have you needed? Please select all that apply.** | | | | | | | | | | |
|  | 1 | Wound dressing | | | | | | | | | |
|  | 2 | Injections | | | | | | | | | |
|  | 3 | Change of feeding tube | | | | | | | | | |
|  | 4 | Change of urinary catheter | | | | | | | | | |
|  | 5 | Physiotherapy | | | | | | | | | |
|  | 6 | Speech Therapy | | | | | | | | | |
|  | 7 | Occupational Therapy | | | | | | | | | |
|  | 8 | Other, please specify: ____ | | | | | | | | | |
|  | 9 | None of the above | | | | | | | | | |
|  | 999 | Don’t know | 777 | Refused | | | | | | | |

| **SECTION L: ACTIVATION IN OWN CARE** | | | | |
| --- | --- | --- | --- | --- |
| **L1** | **How much would you say you know about your health condition(s), for example in terms of symptoms, causes, monitoring, or treatments?** | | | |
|  | 1 | Very little | | |
|  | 2 | Something | | |
|  | 3 | A lot | | |
|  | 999 | Don’t know | 777 | Refused |
| **L2** | **How confident do you feel about your ability to manage any physical ailment?** | | | |
|  | 1 | Not at all confident | | |
|  | 2 | A little confident | | |
|  | 3 | Somewhat confident | | |
|  | 4 | Quite confident | | |
|  | 5 | Extremely confident | | |
|  | 999 | Don’t know | 777 | Refused |
| **L3** | **How often do you have a problem understanding information told to you about your health?** | | | |
|  | 1 | Always | | |
|  | 2 | Often | | |
|  | 3 | Sometimes | | |
|  | 4 | Occasionally | | |
|  | 5 | Never | | |
|  | 999 | Don’t know | 777 | Refused |

| **SECTION M: FUNCTIONAL ASSESSMENT - INSTRUMENTAL ACTIVITIES OF DAILY LIVING** | | | | | |
| --- | --- | --- | --- | --- | --- |
| **The next questions concern your ability to perform slightly complicated tasks.**  **I will mention some activities and ask you whether you find them difficult to perform without help from a person or an assistive device due to your health or physical condition.** | | | | | |
|  | **Do you find it difficult to (M1-M7) alone without the assistance of a person or assistive device due to your health or physical condition?** | **Answer** | | | |
| **M1** | Take public transport to leave home | 1 | Yes | | |
|  |  | 2 | No | | |
|  |  | 3 | I do not perform this activity due to a reason other than my health / physical condition | | |
|  |  | 999 | Don’t know | 777 | Refused |
| **M2** | Leave the home to purchase necessary items or medication | 1 | Yes | | |
|  |  | 2 | No | | |
|  |  | 3 | I do not perform this activity due to a reason other than my health / physical condition | | |
|  |  | 999 | Don’t know | 777 | Refused |
| **M3** | Take care of financial matters such as paying utilities | 1 | Yes | | |
|  |  | 2 | No | | |
|  |  | 3 | I do not perform this activity due to a reason other than my health / physical condition | | |
|  |  | 999 | Don’t know | 777 | Refused |
| **M4** | Dust, clean-up and other light housework | 1 | Yes | | |
|  |  | 2 | No | | |
|  |  | 3 | I do not perform this activity due to a reason other than my health / physical condition | | |
|  |  | 999 | Don’t know | 777 | Refused |
| **M5** | Prepare own meals | 1 | Yes | | |
|  |  | 2 | No | | |
|  |  | 3 | I do not perform this activity due to a reason other than my health / physical condition | | |
|  |  | 999 | Don’t know | 777 | Refused |
| **M6** | Take medication as prescribed | 1 | Yes | | |
|  |  | 2 | No | | |
|  |  | 3 | I do not perform this activity due to a reason other than my health / physical condition | | |
|  |  | 999 | Don’t know | 777 | Refused |
| **M7** | Use the phone | 1 | Yes | | |
|  |  | 2 | No | | |
|  |  | 3 | I do not perform this activity due to a reason other than my health / physical condition | | |
|  |  | 999 | Don’t know | 777 | Refused |

| **SECTION N: FUNCTIONAL ASSESSMENT - ACTIVITIES OF DAILY LIVING** | | | | | |
| --- | --- | --- | --- | --- | --- |
| **The next questions concern your ability to perform basic day-to-day activities.**  **I will mention some activities and ask you whether you find them difficult to perform without help from a person or an assistive device due to your health or physical condition.** | | | | | |
|  | **Do you find it difficult to (N1–N6) alone without the assistance of a person or assistive device due to your health or physical condition?** | **Answer** | | | |
| **N1** | Take a bath / shower | 1 | Yes | | |
|  |  | 2 | No | | |
|  |  | 999 | Don’t know | 777 | Refused |
| **N2** | Dress up | 1 | Yes | | |
|  |  | 2 | No | | |
|  |  | 999 | Don’t know | 777 | Refused |
| **N3** | Walk around the house | 1 | Yes | | |
|  |  | 2 | No | | |
|  |  | 999 | Don’t know | 777 | Refused |
| **N4** | Stand up from or sit down on a bed / chair | 1 | Yes | | |
|  |  | 2 | No | | |
|  |  | 999 | Don’t know | 777 | Refused |
| **N5** | Use the sitting toilet | 1 | Yes | | |
|  |  | 2 | No | | |
|  |  | 999 | Don’t know | 777 | Refused |
| **N6** | Eat | 1 | Yes | | |
|  |  | 2 | No | | |
|  |  | 999 | Don’t know | 777 | Refused |

| **SECTION O: MOBILITY AID** | | | | |
| --- | --- | --- | --- | --- |
| **O1** | **Which of the following aids do you use for walking, either at home or outside? [multiple anwers possible]** | | | |
|  | 1 | Walking stick or cane | | |
|  | 2 | Walking stick or cane, with built-in chair | | |
|  | 3 | Umbrella | | |
|  | 4 | Umbrella with non-slip tip | | |
|  | 5 | Walking frame or walker | | |
|  | 6 | Walking frame or walker with wheels | | |
|  | 7 | Elbow crutches | | |
|  | 8 | Wheelchair | | |
|  | 9 | Support from wall or pillars or railings/bars | | |
|  | 10 | Support from a person | | |
|  | 11 | None of the above | | |
|  | 999 | Don’t know | 777 | Refused |
| **GALI-SCORES** | | | | |
| **O2** | **For at least the past six months, to what extent have you been limited because of a health problem in activities people usually do? Would you say that you have been…** | | | |
|  | 1 | Severely limited | | |
|  | 2 | Limited but not severely | | |
|  | 3 | Not limited at all | | |
|  | 999 | Don’t know | 777 | Refused |

| **UNINTENTIONAL WEIGHT LOSS** | | | | |
| --- | --- | --- | --- | --- |
| **O3** | **In the last year, have you lost more than 5 kilograms of body weight unintentionally (i.e., not due to dieting or exercise)?** | | | |
|  | 1 | Yes | | |
|  | 2 | No | | |
|  | 999 | Don’t know | 777 | Refused |
| **MEDICATION NEED** | | | | |
| **I would like to ask you some questions about your medication intake.** | | | | |
| **O4** | **Do you currently take any prescription medications on a regular basis? By prescription medications, we refer to medicines given by a western doctor such as a General Practitioner (GP), or a polyclinic doctor.** | | | |
|  | 1 | Yes ***[continue to O5]*** | | |
|  | 2 | No ***[continue to O6]*** | | |
|  | 999 | Don’t know | 777 | Refused |
| **O5** | **How many different prescription medications do you take on a regular basis?** | | | |
|  | **__________** different prescription medications | | | |
|  | 999 | Don’t know | 777 | Refused |
| **TOBACCO USE** | | | | |
| **O6** | **Do you smoke tobacco products, including heated tobacco products? Please exclude electronic cigarettes or similar products. (Heated tobacco products refer, for example, to IQOS or HEETS tobacco sticks.)** | | | |
|  | 1 | Yes, daily ***[continue to O6a]*** | | |
|  | 2 | Yes, occasionally ***[continue to O6a]*** | | |
|  | 3 | No, not anymore ***[continue to O6a]*** | | |
|  | 4 | I have never smoked ***[continue to P]*** | | |
|  | 999 | Don’t know | 777 | Refused |
| **O6a** | **Have you ever smoked tobacco products (e.g. cigarettes, cigars, or a pipe) daily or almost daily for at least one year?** | | | |
|  | 1 | Yes ***[continue to O6b]*** | | |
|  | 2 | No ***[continue to P]*** | | |
|  | 999 | Don’t know | 777 | Refused |
| **O6b** | **For how many years have you smoked daily? Please add together all periods in which you smoked daily. If you cannot remember the exact number of years, please provide an estimate.** | | | |
|  | **__________** years | | | |

| **SECTION P: INTERNATIONAL PHYSICAL ACTIVITY QUESTIONNAIRE - SHORT FORM** | | | | |
| --- | --- | --- | --- | --- |
| **We are interested in finding out about the kinds of physical activities that people do as part of their everyday lives. The questions will ask you about the time you spent being physically active in the last 7 days. Please answer each question even if you do not consider yourself to be an active person. Please think about the activities you do at work, as part of your housework, to get from place to place, and in your spare time for recreation, exercise or sport.** | | | | |
| **First, think about all the vigorous activities that you did in the last 7 days. Vigorous physical activities refer to activities that take hard physical effort and make you breathe much harder than normal. Think only about those physical activities that you did for at least 10 minutes at a time.** | | | | |
| **P1a** | **During the last 7 days, on how many days did you do vigorous physical activities like heavy lifting, digging, aerobics, or fast bicycling?** | | | |
|  | _________ days in the last 7 days | | | |
|  | 0 | No vigorous physical activities on any day ***[continue to P2a]*** | | |
|  | 999 | Don’t know ***[continue to P2a]*** | | |
|  | 777 | Refused ***[continue to P2a]*** | | |
| **P1b** | **How much time did you usually spend doing vigorous physical on one of those days?** | | | |
|  |  | _________ hours __________ minutes | | |
|  | 999 | Don’t know | 777 | Refused |
| **Now, think about all the moderate activities that you did in the last 7 days. Moderate activities refer to activities that take moderate physical effort and make you breathe somewhat harder than normal. Think only about those physical activities that you did for at least 10 minutes at a time.** | | | | |
| **P2a** | **During the last 7 days, on how many days did you do moderate physical activities like carrying light loads, bicycling at a regular pace, or doubles tennis? Do not include walking.** | | | |
|  | _________ days in the last 7 days | | | |
|  | 0 | No moderate physical activities on any day ***[continue to P3a]*** | | |
|  | 999 | Don’t know ***[continue to P3a]*** | | |
|  | 777 | Refused ***[continue to P3a]*** | | |
| **P2b** | **How much time did you usually spend doing moderate physical activities on one of those days?** | | | |
|  | _________ hours __________ minutes | | | |
|  | 999 | Don’t know | | |
|  | 777 | Refused | | |
| **Now think about the time you spent walking in the last 7 days. This includes walking at work and at home, walking to travel from place to place, and any other walking that you have done solely for recreation, sport, exercise, or leisure.** | | | | |
| **P3a** | **During the last 7 days, on how many days did you walk for at least 10 minutes at a time?** | | | |
|  | _________ days in the last 7 days | | | |
|  | 0 | No walking ***[continue to P4]*** | | |
|  | 999 | Don’t know ***[continue to P4]*** | | |
|  | 777 | Refused ***[continue to P4]*** | | |
| **P3b** | **How much time did you usually spend walking on one of those days?** | | | |
|  | _________ hours__________ minutes | | | |
|  | 999 | Don’t know | | |
|  | 777 | Refused | | |
| **The last question is about the time you spent sitting on weekdays (that is, not including Saturday and Sunday) during the last 7 days. Include time spent at work, at home, while doing course work and during leisure time. This may include time spent sitting at a desk, visiting friends, reading, or sitting or lying down to watch television.** | | | | |
| **P4** | **During the last 7 days and not counting Saturday and Sunday, how much time did you spend sitting on one of those days?** | | | |
|  | _________ hours __________ minutes | | | |
|  | 999 | Don’t know | | |
|  | 777 | Refused | | |

| **SECTION Q: ANXIETY & DEPRESSION (PHQ-4)** | | | | | | | | | | | | | | | | | | | | | | | | | | | | | | | |
| --- | --- | --- | --- | --- | --- | --- | --- | --- | --- | --- | --- | --- | --- | --- | --- | --- | --- | --- | --- | --- | --- | --- | --- | --- | --- | --- | --- | --- | --- | --- | --- |
| **Over the last two weeks, how often have you felt or thought the following?** | | | | | | | | | | | | | | | | | | | | | | | | | | | | | | | |
|  |  | | | | | | | **Not at all** | | | **Several days** | | | | **More than half of the days** | | | | | **Nearly every day** | | | | **Don’t know** | | | | | **Refused** | | |
| **Q1** | Feeling nervous, anxious, or on edge | | | | | | | 0 | | | 1 | | | | 2 | | | | | 3 | | | | 999 | | | | | 777 | | |
| **Q2** | Not being able to stop or control worrying | | | | | | | 0 | | | 1 | | | | 2 | | | | | 3 | | | | 999 | | | | | 777 | | |
| **Q3** | Little interest or pleasure in doing things | | | | | | | 0 | | | 1 | | | | 2 | | | | | 3 | | | | 999 | | | | | 777 | | |
| **Q4** | Feeling down, depressed, or hopeless | | | | | | | 0 | | | 1 | | | | 2 | | | | | 3 | | | | 999 | | | | | 777 | | |
| **During the past week, to what extent has the following been true for you?** | | | | | | | | | | | | | | | | | | | | | | | | | | | | | | | |
|  | | | | | | | | **None/ rarely** | | | | **Sometimes** | | | | | **Often** | | | | | **Don’t know** | | | | | | **Refused** | | | |
| **Q5** | I felt that everything I did was an effort | | | | | | | 0 | | | | 1 | | | | | 2 | | | | | 999 | | | | | | 777 | | | |
| **Q6** | I could not get “going” | | | | | | | 0 | | | | 1 | | | | | 2 | | | | | 999 | | | | | | 777 | | | |
| **CONTROL BELIEFS** | | | | | | | | | | | | | | | | | | | | | | | | | | | | | | | |
|  |  | | | | | | **Strongly disagree** | | | **Disagree** | | | | **Neutral** | | | | **Agree** | | | **Strongly agree** | | | | **Don’t know** | | | | | **Refused** | |
| **Q7** | It's up to me to arrange  for all the good things in  my life. | | | | | | 1 | | | 2 | | | | 3 | | | | 4 | | | 5 | | | | 999 | | | | | 777 | |
| **Q8** | When I get what I want,  it is usually because I  have worked hard for it. | | | | | | 1 | | | 2 | | | | 3 | | | | 4 | | | 5 | | | | 999 | | | | | 777 | |
| **Q9** | I can make sure that good  things come my way. | | | | | | 1 | | | 2 | | | | 3 | | | | 4 | | | 5 | | | | 999 | | | | | 777 | |
| **WELL-BEING** | | | | | | | | | | | | | | | | | | | | | | | | | | | | | | | |
| **Overall, how satisfied are you with your life at present?** | | | | | | | | | | | | | | | | | | | | | | | | | | | | | | | |
| **Q10** | Completely dissatisfied | | | |  | | | | | | | | | | | | | | | | | | | | | | Completely satisfied | | | | |
|  | 0 | | 1 | 2 | | 3 | | | 4 | | | | 5 | | | 6 | | | 7 | | | | 8 | | | 9 | | | | | 10 |
|  | 999 | Don’t know | | | | | | | | | | | | | | | | | | | | | | | | | | | | | |
|  | 777 | Refused | | | | | | | | | | | | | | | | | | | | | | | | | | | | | |

| **SECTION R: BASIC ATTRIBUTES** | | | | | | | | | | | | | | | | |
| --- | --- | --- | --- | --- | --- | --- | --- | --- | --- | --- | --- | --- | --- | --- | --- | --- |
| **First, we would like to ask you some questions about yourself.** | | | | | | | | | | | | | | | | |
| **R1** | **What is your gender? (Select all that apply.)** | | | | | | | | | | | | | | | |
|  | 1 | Female | | | | | | | | | | | | | | |
|  | 2 | Male | | | | | | | | | | | | | | |
|  | 3 | Cis | | | | | | | | | | | | | | |
|  | 4 | Endo* | | | | | | | | | | | | | | |
|  | 5 | Inter* | | | | | | | | | | | | | | |
|  | 6 | Non-binary | | | | | | | | | | | | | | |
|  | 7 | I don’t know | | | | | | | | | | | | | | |
|  | 8 | Trans* | | | | | | | | | | | | | | |
|  | 9 | Prefer to self-describe: **______________** | | | | | | | | | | | | | | |
|  | 10 | Refused | | | | | | | | | | | | | | |
| **R2** | **Which country were you born in?** | | | | | | | | | | | | | | | |
|  | Drop- down- menu with countries | | | | | | | Country not listed: **_____________** | | | | | | 777 | Refused | |
|  |  | | ***Filter: If born in Germany:*** | | | | | | | | | | | | | |
|  | **Have you ever lived in the former German Democratic Republic (GDR)?** | | | | | | | | | | | | | | | |
|  | 1 | | Yes | | | | | | | | | | | | | |
|  | 2 | | No | | | | | | | | | | | | | |
|  | 999 | | Don’t know | | | | 777 | | | | Refused | | | | | |
| **R3** | **In which country were your parents born?** | | | | | | | | | | | | | | | |
|  | Parent 1/Mother | | | | Drop- down- menu with countries | | | | | | | | Country not listed: **_____________** | 777 | | Refused |
|  | Parent 2/Father | | | | Drop- down- menu with countries | | | | | | | | Country not listed: **_____________** | 777 | | Refused |
| **R4** | **Is German your native language?** | | | | | | | | | | | | | | | |
|  | 1 | | Yes | | | | | | | | | | | | | |
|  |  | | **Do you have any other native languages?** | | | | | | | | | | | | | |
|  |  |  | 1 | Yes: **______________** | | | | | | | | | | | | |
|  |  |  | 2 | No | | | | | | | | | | | | |
|  |  |  | 999 | Don’t know | | | | | | | 777 | Refused | | | | |
|  | 2 | | No | | | | | | | | | | | | | |
|  |  | | **How would you rate your German skills?** | | | | | | | | | | | | | |
|  |  |  | 1 | Very good | | | | | | | | | | | | |
|  |  |  | 2 | Good | | | | | | | | | | | | |
|  |  |  | 3 | Moderate | | | | | | | | | | | | |
|  |  |  | 4 | Poor | | | | | | | | | | | | |
|  |  |  | 0 | Very Poor | | | | | | | | | | | | |
|  |  |  | 999 | Don’t know | | | | | | | 777 | Refused | | | | |
|  |  |  | **What is your native language?** | | | | | | | | | | | | | |
|  |  |  | **______________** | | | | | | | | | | | | | |
|  |  |  | 999 | Don’t know | | | | | | | 777 | Refused | | | | |
| **R5** | **What is the highest level of general education you have completed?** | | | | | | | | | | | | | | | |
|  | 1 | | No qualification yet (currently in school) | | | | | | | | | | | | | |
|  | 2 | | No general school-leaving qualification | | | | | | | | | | | | | |
|  | 3 | | School-leaving qualification after up to 7 years of schooling | | | | | | | | | | | | | |
|  | 4 | | Lower secondary school-leaving certificate (e.g., Hauptschule / Volksschule / Polytechnic Secondary School after grade 8 or 9) | | | | | | | | | | | | | |
|  | 5 | | Intermediate secondary school-leaving certificate (e.g., Realschule / Mittlere Reife / Polytechnic Secondary School after grade 10) | | | | | | | | | | | | | |
|  | 6 | | Higher education entrance qualification (e.g., Abitur, subject-specific higher education entrance qualification, or university of applied sciences entrance qualification) | | | | | | | | | | | | | |
|  | 7 | | Other qualification: **__________** | | | | | | | | | | | | | |
|  | 999 | | Don’t know | | | | 777 | | Refused | | | | | | | |
| **R6** | **What is the highest vocational training or university/technical college degree you have?** | | | | | | | | | | | | | | | |
|  | 1 | | No vocational qualification and not in vocational training | | | | | | | | | | | | | |
|  | 2 | | Completed vocational training (apprenticeship) | | | | | | | | | | | | | |
|  | 3 | | Completed vocational school education (vocational school , business school ) | | | | | | | | | | | | | |
|  | 4 | | Completed training at a technical college, master craftsman school , technical school , vocational school , or technical academy | | | | | | | | | | | | | |
|  | 5 | | University studies, not completed | | | | | | | | | | | | | |
|  | 6 | | Completed (technical) university degree: **_________** | | | | | | | | | | | | | |
|  | 999 | | Don’t know | | | 777 | | | | Refused | | | | | | |
|  | *If completed (technical) university degree* | | | | | | | | | | | | | | | |
|  | 7 | | Bachelor’s degree | | | | | | | | | | | | | |
|  | 8 | | Master’s degree | | | | | | | | | | | | | |
|  | 9 | | Diploma (German “Diplom”) | | | | | | | | | | | | | |
|  | 10 | | Magister degree | | | | | | | | | | | | | |
|  | 11 | | State examination (Staatsexamen) | | | | | | | | | | | | | |
|  | 12 | | Doctoral degree (PhD) | | | | | | | | | | | | | |
|  | 13 | | Habilitation (postdoctoral qualification) | | | | | | | | | | | | | |
|  | 14 | | Other qualification, namely: **__________** | | | | | | | | | | | | | |
|  | 999 | | Don’t know | | | | 777 | | Refused | | | | | | | |
| **INCOME** | | | | | | | | | | | | | | | | |
| **Do you feel that you have adequate income to meet your expenses per month?** | | | | | | | | | | | | | | | | |
| **R7** | 1 | | Enough money, with some left over | | | | | | | | | | | | | |
|  | 2 | | Just enough money, no difficulty | | | | | | | | | | | | | |
|  | 3 | | Some difficulty to meet expenses | | | | | | | | | | | | | |
|  | 4 | | Much difficulty to meet expenses | | | | | | | | | | | | | |
|  | 999 | | Don’t know | | | | 777 | | Refused | | | | | | | |

| **MACARTHUR SCALE OF SUBJECTIVE SOCIAL STATUS – ADULT VERSION** | |
| --- | --- |
| **R8** | **Think of this ladder as representing where people stand in Germany. At the top of the ladder are the people who are the best off – those who have the most money, the most education, and the most respected jobs. At the bottom are the people who are the worst off – those who have the least money, least education, the least respected jobs, or no job. The higher up you are on this ladder, the closer you are to the people at the very top; the lower you are, the closer you are to the people at the very bottom.**  **Where would you place yourself on this ladder?**  **Please place a large “X” on the rung where you think you stand at this time in your life relative to other people in Germany.**   |

| **SECTION S: FRAILTY SPECIFIC QUESTIONS BASED ON FRIED’S SCALE** | | | | | | | | | | | | | |
| --- | --- | --- | --- | --- | --- | --- | --- | --- | --- | --- | --- | --- | --- |
| **HANDGRIP STRENGTH** | | | | | | | | | | | | | |
| **S1** | **Before we begin, I would like to make sure it is safe for you to do this test. Have you had surgery or experienced any swelling, inflammation, severe pain, paralysis or injury in one or both hands within the last 6 months?** | | | | | | | | | | | | |
|  | 1 | | Yes ***[Go to S2]*** | | | | | | | | | | |
|  | 2 | | No ***[Go to S3]*** | | | | | | | | | | |
| **S2** | **In which hand have you had surgery or experienced any swelling, inflammation, severe pain, paralysis, or injury in the last 6 months?** | | | | | | | | | | | | |
|  | 1 | | Both hands ***[Do not complete this measure and go to S11]*** | | | | | | | | | | |
|  | 2 | | Left hand only ***[Do not perform test on left hand]*** | | | | | | | | | | |
|  | 3 | | Right hand only ***[Do not perform test on right hand]*** | | | | | | | | | | |
| **S3** | **Do you understand the directions for this test?** | | | | | | | | | | | | |
|  | 1 | | Yes | | | | | | | | | | |
|  | 2 | | No ***[Do not complete this measure and go to S11]*** | | | | | | | | | | |
| **S4** | **Do you feel it would be safe for you to do this test?** | | | | | | | | | | | | |
|  | 1 | | Yes | | | | | | | | | | |
|  | 2 | | No ***[Do not complete this measure and go to S11]*** | | | | | | | | | | |
| **S5** | **Which hand do you normally use?** | | | | | | | | | | | | |
|  | 1 | | Right hand | | | | | | | | | | |
|  | 2 | | Left hand | | | | | | | | | | |
|  | 3 | | Both hands equally dominant | | | | | | | | | | |
| **INSTRUCTIONS FOR ADMINISTERING THE HAND STRENGTH TEST**   a) Suggest respondent removes rings or other hand jewelry. b) Position the Respondent correctly, standing with arm at side. Adjust dynamometer to hand size by adjusting the knob. c) Reset arrow at zero. d) Explain the procedure once again. e) Let Respondent have a practice with their dominant hand and then reset the marker. f) Subject to feasibility established in S2, conduct the test on the dominant hand first, followed by the non-dominant hand. g) Repeat, for a total of two tests on each hand. h) Record measurements to the nearest 0.5 kilogram in the table below (e.g., 10.5 kg). | | | | | | | | | | | | | |
| *[Instruction for Interviewer: Record measurements to the nearest 0.5 kilogram (example: record 10.5 kg) in table below]* | | | | | | | | | | | | | |
| **S6** | **Measurement** | | | **Left Hand** | | **Right Hand** | | | | **Eligible hand/s completed** | | | |
|  | 1. | | | : kg | | : kg | | | | 1 Yes | | | 2 No |
|  | 2. | | | : kg | | : kg | | | | 1 Yes | | | 2 No |
| *[Instructions for Interviewer:*    *If BOTH hands are eligible:*     - *If all four measurements in S6 are completed, then go to S8* - *If only 1 or 2 or 3 measurements in S6 are completed, then go to S7 and then go to S8* - *If none of the measurements in S6 are completed, then go to S7 and then go to  S11*   *If ONLY ONE hand is eligible:*     - *If both measurements in S6 are completed, then go to S8* - *If only 1 measurement in S6 is completed, then go to S7 and then go to S8* - *If none of the measurements in S6 are completed, then go to S7 and then go to S11]* | | | | | | | | | | | | | |
| **S7** | ***[Answered by Interviewer: Record why the hand strength test was not completed] [MA]*** | | | | | | | | | | | | |
|  | 1 | Respondent felt it would not be safe | | | | | | | | | | | |
|  | 2 | Interviewer felt it would not be safe | | | | | | | | | | | |
|  | 3 | Respondent refused or was not willing to complete the test | | | | | | | | | | | |
|  | 4 | Respondent tried but was unable to complete test | | | | | | | | | | | |
|  | 5 | Respondent did not understand the instructions | | | | | | | | | | | |
|  | 6 | Others (Please specify: _______) | | | | | | | | | | | |
| **S8** | ***[Answered by Interviewer: Record how much effort the respondent gave to this test.]*** | | | | | | | | | | | | |
|  | 1 | Respondent gave full effort | | | | | | | | | | | |
|  | 2 | Respondent was prevented from giving full effort by illness, pain, or other symptoms or  discomforts | | | | | | | | | | | |
|  | 3 | Respondent did not appear to give full effort, but no obvious reason for this | | | | | | | | | | | |
| **S9** | ***[Answered by Interviewer: Record what the respondent’s position was for this test.]*** | | | | | | | | | | | | |
|  | 1 | Standing | | | | 2 | Sitting | | 3 | | Lying down | | |
| **S10** | ***[Answered by Interviewer: Record whether the respondent rested his/her arm on a support while***  ***performing the test.]*** | | | | | | | | | | | | |
|  | 1 | Yes | | | | 2 | No | | | | | | |
| **WALKING SPEED** | | | | | | | | | | | | | |
| *[Equipment needed: Stopwatch, distance measure, masking tape]*  ***Next, I would like to test whether you can walk a very short distance comfortably. You can use a walking stick or other aid like a walker or crutches, if necessary.*** | | | | | | | | | | | | | |
| **S11** | **First, I want to make sure it is safe to carry out the test. Do you have any problems from recent surgery, injury, or other health conditions that currently prevent you from walking on your own without human assistance?** | | | | | | | | | | | | |
|  | 1 | I am able to walk on my own without human assistance, using a walking stick or other aid if necessary ***[Go to S12]*** | | | | | | | | | | | |
|  | 2 | I am unable to walk on my own without human assistance because of recent surgery  *[****Do not complete this measure and End]*** | | | | | | | | | | | |
|  | 3 | I am unable to walk on my own without human assistance because of injury  *[****Do not complete this measure and End]*** | | | | | | | | | | | |
|  | 4 | I am unable to walk on my own without human assistance because of other health condition(s) *[****Do not complete this measure and End]*** | | | | | | | | | | | |
| **Now let us find a place where we can conduct the test. We will need a clear space about 2.5 meters long in a non-carpeted area, if possible. I will mark the start and finish lines for the walk with masking tape.**  *[Instruction for Interviewer: Set up the course (2.5 Meters or 98.5 Inches).]*  **This is our walking course. I am going to time you as you walk the length of the course. I will ask you to walk the course two times. I will walk alongside you the whole time during the test. You will start by lining your feet up at the starting point.**  *[Instruction for Interviewer: Demonstrate the test.]* | | | | | | | | | | | | | |
| **S12** | **Do you understand the directions for this test?** | | | | | | | | | | | | |
|  | 1 | Yes | | | | | | | | | | | |
|  | 2 | No ***[Do not complete this measure and End]*** | | | | | | | | | | | |
| **S13** | **Do you feel it would be safe for you to do this test*?*** | | | | | | | | | | | | |
|  | 1 | Yes ***[Go to S14]*** | | | | | | | | | | | |
|  | 2 | No ***[Do not complete this measure and End]*** | | | | | | | | | | | |
| **INSTRUCTIONS FOR WALKING SPEED TEST**  a) Ensure that the respondent is wearing appropriate footwear at this point. If not, ask respondent to change shoes or to put shoes on.  b) Instruct respondent to stand with his/her feet lined up to the front of the tape.  c) Say the following to the respondent: **I am going to time you as you walk the course at your normal pace. I will be asking you to walk the course at your usual pace a total of two times. I will walk alongside you the whole time during the test. I would like you to stand here with your feet lined up. Start walking when I say “Begin”. Walk all the way past the other end of the tape before you stop. Are you ready to go now? Begin.**  d) **Start the stopwatch when** either foot is placed down on the floor across the start line.  e) Walk behind and slightly to the side of the respondent.  f) **Stop the stopwatch when** Respondent’s foot crosses the tape and touches the floor. Record the time to the nearest minutes: seconds. hundredths (e.g., 0:04.52) in the table below.  g) Repeat the measurement. Say the following: **Now I want you to repeat the walk. Remember to walk at your usual pace and go all the way past the other end of the course. I would like you to stand here with your feet lined up. Start walking when I say “Begin”. Are you ready to go now? Begin.**  h) **Start the stopwatch when** either foot is placed down on the floor across the start line.  i) Walk behind and slightly to the side of the respondent.  j) **Stop the stopwatch when** respondent’s foot crosses the tape and touches the floor. Record the time to the nearest minutes: seconds. hundredths (e.g., 0:04.52) in the table below. | | | | | | | | | | | | | |
| **[*Instruction for Interviewer*: Record measurements to the nearest minutes: seconds. hundredths (example: record 0:04.52) in the table below.]** | | | | | | | | | | | | | |
| **S14** | **Measurement** | | | | **Walking Speed Time** | | | **Completed** | | | | | |
|  | 1. | | | | **_____ : ____ ____.____ ___**  (minutes) (seconds) (hundredths) | | | **1 Yes** | | | | **2 No** | |
|  | 2. | | | | **_____ : ____ ____.____ ___**  (minutes) (seconds) (hundredths) | | | **1 Yes** | | | | **2 No** | |
| ***[Instruction for Interviewer:***  ***1. If both 1st and 2nd measurements in S14 are completed, then go to S16***  ***2. If only the 1st measurement in S14 is completed, then go to S15 and then Go to S16***  ***3. If none of the measurements in S14 are completed, then go to S15 and then END. ]*** | | | | | | | | | | | | | |
| **S15** | ***[Answered by Interviewer: Record why the walking speed test was not completed] [MA]*** | | | | | | | | | | | | |
|  | 1 | Respondent felt it would not be safe | | | | | | | | | | | |
|  | 2 | Interviewer felt it would not be safe | | | | | | | | | | | |
|  | 3 | Respondent refused or was not willing to complete the test | | | | | | | | | | | |
|  | 4 | Respondent tried but was unable to complete test | | | | | | | | | | | |
|  | 5 | Respondent did not understand the instructions | | | | | | | | | | | |
|  | 6 | No suitable space available | | | | | | | | | | | |
|  | 7 | Others (please specify): _______ | | | | | | | | | | | |
| **S16** | ***[Answered by Interviewer: Record type of aid used]:*** | | | | | | | | | | | | |
|  | 1 | None | | | | | | | | | | | |
|  | 2 | Walking stick, cane or umbrella | | | | | | | | | | | |
|  | 3 | Elbow crutches | | | | | | | | | | | |
|  | 4 | Walking frame | | | | | | | | | | | |
|  | 5 | Other (please specify): _______ | | | | | | | | | | | |
| **S17** | ***[Answered by Interviewer: How compliant was respondent during this measurement]:*** | | | | | | | | | | | | |
|  | 1 | Respondent was fully compliant | | | | | | | | | | | |
|  | 2 | Respondent was prevented from fully complying due to illness, pain, or other symptoms or discomforts | | | | | | | | | | | |
|  | 3 | Respondent was not fully compliant, but no obvious reason for this | | | | | | | | | | | |
