# Supplementary material for: Which aspects of continuity of care in primary care are associated with better self-rated health? A cross-sectional study among older adults in Berlin
Source: BMC Prim Care. 2026 Sep 24;27:339. doi: 10.1186/s12875-026-03566-1 (PMC13602587; doi:10.1186/s12875-026-03566-1)
Supplement: Supplementary file 2 [file 12875_2026_3566_MOESM2_ESM.docx]

| **Table 4**  *Linear regression of self-rated health with alternative morbidity score* | | |
| --- | --- | --- |
| Predictor | Model 3 | |
|  | b | 95% CI |
| GP site continuity | -0.021 | -0.054, 0.012 |
| GP doctor continuity | 0.008** | 0.002, 0.015 |
| Age | -0.01* | -0.019, -0.002 |
| Gender (male) | -0.13* | -0.25, -0.009 |
| SSS | 0.054** | 0.017, 0.092 |
| Morbidity ratio | -3.437*** | -4.012, -2.862 |
| Symptoms of depression | -0.396** | -0.651, -0.142 |
| Symptoms of anxiety | -0.134 | -0.416, 0.149 |
| *n* | 748 | |
| R² | 0.244 | |
| Adjusted R² | 0.236 | |
| AIC | 1807.97 | |
| *Note****.*** b = unstandardized regression coefficient. 95% CI = 95% confidence interval. Partial *R²* values are reported as measures of effect size. Significance levels correspond to the t-test of the regression coefficients. **p* < .05, ***p* < .01, *** *p* < .001. GP = general practitioner. SSS = subjective social status. AIC = akaike information criterion. | | |
