# Supplementary material for: Which aspects of continuity of care in primary care are associated with better self-rated health? A cross-sectional study among older adults in Berlin
Source: BMC Prim Care. 2026 Sep 24;27:339. doi: 10.1186/s12875-026-03566-1 (PMC13602587; doi:10.1186/s12875-026-03566-1)
Supplement: Supplementary file 3 [file 12875_2026_3566_MOESM3_ESM.docx]

| **Table 5**  *Linear regression of self-rated health excluding participants with GP doctor continuity > 40 years (n = 9 excluded)* | | |
| --- | --- | --- |
| Predictor | Model 3 | |
|  | b | 95% CI |
| GP site continuity | -0.023 | -0.056, 0.01 |
| GP doctor continuity | 0.01** | 0.003, 0.017 |
| Age | -0.01* | -0.018, -0.001 |
| Gender (male) | -0.127* | -0.247, -0.006 |
| SSS | 0.056** | 0.019, 0.093 |
| Morbidity ratio | -0.132*** | -0.154, -0.11 |
| Symptoms of depression | -0.401** | -0.655, -0.146 |
| Symptoms of anxiety | -0.128 | -0.411, 0.155 |
| *n* | 739 | |
| R² | 0.244 | |
| Adjusted R² | 0.236 | |
| AIC | 1787.07 | |
| *Note****.*** b = unstandardized regression coefficient. 95% CI = 95% confidence interval. Partial *R²* values are reported as measures of effect size. Significance levels correspond to the t-test of the regression coefficients. **p* < .05, ***p* < .01, *** *p* < .001. GP = general practitioner. SSS = subjective social status. AIC = akaike information criterion. | | |
