# Supplementary material for: Which aspects of continuity of care in primary care are associated with better self-rated health? A cross-sectional study among older adults in Berlin
Source: BMC Prim Care. 2026 Sep 24;27:339. doi: 10.1186/s12875-026-03566-1 (PMC13602587; doi:10.1186/s12875-026-03566-1)
Supplement: Supplementary file 4 [file 12875_2026_3566_MOESM4_ESM.docx]

| **Table 6**  *Linear regression of self-rated health with effect-coded predictor neighborhood* | | |
| --- | --- | --- |
| Predictor | Model 3 | |
|  | b | 95% CI |
| GP site continuity | -0.018 | -0.053, 0.016 |
| GP doctor continuity | **0.008*** | **0.002, 0.015** |
| Neighbourhood: Marzahn-Hellersdorf | -0.018 | -0.19, 0.154 |
| Neighbourhood: Reinickendorf | 0.09 | -0.1, 0.279 |
| GP site continuity × Marzahn-Hellersdorf | 0.019 | -0.026, 0.064 |
| GP site continuity × Reinickendorf | -0.041 | -0.089, 0.008 |
| GP doctor continuity × Marzahn-Hellersdorf | 0 | -0.008, 0.009 |
| GP doctor continuity × Reinickendorf | 0.001 | -0.008, 0.01 |
| Age | **-0.01*** | **-0.019, -0.002** |
| Gender (male) | **-0.129*** | **-0.249, -0.009** |
| SSS | **0.054**** | **0.017, 0.091** |
| Lifetime morbidity | **-0.132***** | **-0.154, -0.109** |
| Symptoms of depression | **-0.399**** | **-0.654, -0.143** |
| Symptoms of anxiety | -0.134 | -0.417, 0.149 |
| *n* | 748 | |
| R² | 0.25 | |
| Adjusted R² | 0.236 | |
| AIC | 1814.23 | |
| *Note****.*** b = unstandardized regression coefficient. 95% CI = 95% confidence interval. Significance levels correspond to the t-test of the regression coefficients. **p* < .05, ***p* < .01, *** *p* < .001. GP = general practitioner. SSS = subjective social status. AIC = akaike information criterion. Residence was effect-coded; coefficients represent deviations from the grand mean. Neighbourhood Spandau is the omitted category and is represented by the negative sum of the coefficients. | | |
