# Supplementary material for: Which aspects of continuity of care in primary care are associated with better self-rated health? A cross-sectional study among older adults in Berlin
Source: BMC Prim Care. 2026 Sep 24;27:339. doi: 10.1186/s12875-026-03566-1 (PMC13602587; doi:10.1186/s12875-026-03566-1)
Supplement: Supplementary file 5 [file 12875_2026_3566_MOESM5_ESM.docx]

| **Table 7**  *Comparison of participants included and excluded in the final linear regression model due to missing data* | | | | |
| --- | --- | --- | --- | --- |
|  | **Excluded** | **Included** |  |  |
| Characteristic | *n* = 89 | *n* = 748 | *p* |  |
| **Age** *M (SD)* | 76.15 (7.05) | 75.69 (6.88) | 0.586 | |
| **Gender** *n (%)* |  |  | 0.410 | |
| Female | 55 (65.48) | 451 (60.29) |  | |
| Male | 29 (34.52) | 297 (39.71) |  | |
| **Neighborhood** *n (%)* |  |  | 0.124 | |
| Marzahn-Hellersdorf | 28 (31.46) | 281 (37.57) |  | |
| Reinickendorf | 26 (29.21) | 253 (33.82) |  | |
| Spandau | 35 (39.33) | 214 (28.61) |  | |
| **SSS** *M (SD)* | 6.71 (1.61) | 6.49 (1.63) | 0.437 | |
| **Lifetime morbidity** *M (SD)* | 4.49 (2.93) | 4.81 (2.79) | 0.299 | |
| **Symptoms of depression** *n (%)* |  |  | 0.810 | |
| No | 69 (94.52) | 695 (92.91) |  | |
| Yes | 4 (5.48) | 53 (7.09) |  | |
| **Symptoms of anxiety** *n (%)* |  |  | 0.789 | |
| No | 71 (95.95) | 707 (94.52) |  | |
| Yes | 3 (4.05) | 41 (5.48) |  | |
| **GP site continuity** *n (%)* |  |  | 0.122 | |
| Other care place | 13 (21.67) | 96 (12.83) |  | |
| Less than 6 months | 1 (1.67) | 5 (0.67) |  | |
| 6 months till 1 year | 3 (5.00) | 23 (3.07) |  | |
| 1 year to 2 years | 1 (1.67) | 33 (4.41) |  | |
| 2 years to 5 years | 9 (15.00) | 88 (11.76) |  | |
| 5 years or more | 33 (55.00) | 503 (67.25) |  | |
| **GP doctor continuity** *M (SD)* | 3.50 (8.29) | 7.67 (10.10) | < 0.001 | |
| **Self-rated health** *n (%)* |  |  | 0.309 | |
| Poor | 14 (15.91) | 68 (9.09) |  | |
| Moderate | 27 (30.68) | 251 (33.56) |  | |
| Good | 37 (42.05) | 311 (41.58) |  | |
| Very good | 9 (10.23) | 94 (12.57) |  | |
| Excellent | 1 (1.14) | 24 (3.21) |  | |
| *Note. N* = 837. M = mean. SD = standard deviation. SSS = subjective social status. Lifetime morbidity = mean of chronic conditions with SD. GP = general practitioner. *p* = p value. P values were calculated using the Wilcoxon rank-sum test for continuous and ordinal variables and Fisher’s exact test for categorial variables. | | | | |
